# Changes in Human Fecal Microbiota Due to Chemotherapy Analyzed by TaqMan-PCR, 454 Sequencing and PCR-DGGE Fingerprinting

**DOI:** 10.1371/journal.pone.0028654

**Published:** 2011-12-14

**Authors:** Jutta Zwielehner, Cornelia Lassl, Berit Hippe, Angelika Pointner, Olivier J. Switzeny, Marlene Remely, Elvira Kitzweger, Reinhard Ruckser, Alexander G. Haslberger

**Affiliations:** 1 Department of Nutritional Sciences, Vienna, Austria; 2 Sozialmedizinisches Zentrum Ost, Vienna, Austria; Charité, Campus Benjamin Franklin, Germany

## Abstract

**Background:**

We investigated whether chemotherapy with the presence or absence of antibiotics against different kinds of cancer changed the gastrointestinal microbiota.

**Methodology/Principal Findings:**

Feces of 17 ambulant patients receiving chemotherapy with or without concomitant antibiotics were analyzed before and after the chemotherapy cycle at four time points in comparison to 17 gender-, age- and lifestyle-matched healthy controls. We targeted 16S rRNA genes of all bacteria, *Bacteroides*, bifidobacteria, *Clostridium* cluster *IV* and *XIVa* as well as *C. difficile* with TaqMan qPCR, denaturing gradient gel electrophoresis (DGGE) fingerprinting and high-throughput sequencing. After a significant drop in the abundance of microbiota (p = 0.037) following a single treatment the microbiota recovered within a few days. The chemotherapeutical treatment marginally affected the *Bacteroides* while the *Clostridium* cluster *IV* and *XIVa* were significantly more sensitive to chemotherapy and antibiotic treatment. DGGE fingerprinting showed decreased diversity of *Clostridium* cluster *IV* and *XIVa* in response to chemotherapy with cluster *IV* diversity being particularly affected by antibiotics. The occurrence of *C. difficile* in three out of seventeen subjects was accompanied by a decrease in the genera *Bifidobacterium*, *Lactobacillus*, *Veillonella* and *Faecalibacterium prausnitzii*. *Enterococcus faecium* increased following chemotherapy.

**Conclusions/Significance:**

Despite high individual variations, these results suggest that the observed changes in the human gut microbiota may favor colonization with *C.difficile* and *Enterococcus faecium*. Perturbed microbiota may be a target for specific mitigation with safe pre- and probiotics.

## Introduction

The human intestinal ecosystem can be pictured as a microbial organ within a host organism involving a dynamic interplay between food, host cells and microbes [1]. The microbiota plays several significant roles in the digestion of food, energy regulation, generation of short-chain fatty acids, vitamin synthesis, prevention of colonization by pathogens and protection against cell injury [2], [3], [4]. Moreover, the gut microbiota influences the host by directing intestinal epithelial cell proliferation and differentiation, pH, and the development of the immune system [1]. Recent culture-independent molecular studies on healthy individuals have shown that the intestinal microbiota is specific to the host [5] and resilient to modifications over time, as it is able to form an alternative stable state after disruption [6], [7]. A healthy microbiota contains a balanced composition of many classes of bacteria [8]. The fecal microbiota is dominated by three groups of anaerobic bacteria: the *Clostridium coccoides* group -clostridial cluster *XIVa* (reclassified as *Blautia coccoides* [9]), the *Clostridium leptum* group - *Clostridium* cluster *IV*, and the *Bacteroides* [10], [11]. All three groups are known to positively affect the gut health through nutrient absorption, production of short chain fatty acids (SCFAs) and epithelial cell maturation [12], [13]. Moreover, the subgroup bifidobacteria seems to be an important part of the gastrointestinal tract (GI) microbiota, being involved in the prevention of atopic disease, obesity and insulin resistance via enhanced barrier function of the gut epithelium [14].

To prevent the invasion of endogenous bacteria from oral cavity and the GI tract into the blood stream, three defense mechanisms are considered to be relevant: innate immunity, mechanical mucosal barrier, and colonization resistance [15]. However, chemotherapy damages the rapidly generated mucosal cells of the GI and the use of antibiotics disrupts the ecological balance, allowing pathogens such as *Clostridium difficile* to grow [16], [17]. This bacterium is thought to be the causative agent in up to 20% of antibiotic-associated diarrhea (AAD) cases [18]. It is evident that the intestinal microbial ecosystem has an important but incompletely defined role in mucosal protection [19].

Mucositis is a major oncological problem, caused by the cytotoxic effects of cancer chemotherapy and radiotherapy [20]. Approximately 40% of patients receiving standard dose chemotherapy and up to 100% of patients receiving high dose chemotherapy and stem cell or bone marrow transplantation suffer from abdominal pain, ulceration, bloating and vomiting [21], [22]. Although gastrointestinal disturbances (mucositis, diarrhea and constipation) and immunosuppression are well recognized side-effects of cancer treatment, very little research has been conducted into the underlying mechanisms and the changes in the composition of the microbiota. Because of these changes, nutrient absorption and other intestinal functions involving the microbiota may also be altered [23].

For this reason, we investigated shifts in fecal microbiota of patients receiving cancer chemotherapy with or without antibiotics in comparison to healthy control individuals. Prescription of antibiotics may become necessary in some individuals due to bacterial infection [24]. Samples were taken at four time points before and after chemotherapy to study changes in fecal microbiota over the course of time. In this study, we aimed to clarify how chemotherapy agents influence total fecal bacteria, *Bacteroides*, bifidobacteria, *Clostridium* cluster *IV*, *Clostridium* cluster *XIVa* and *C. difficile* using culture-independent methods assessing abundance and diversity. Four samples were also analyzed with 454 high-throughput sequencing.

## Results

### PCR-DGGE fingerprinting analysis shows decreased diversity of *Clostridium* clusters *IV* and *XIVa* in response to medical treatment compared to healthy individuals

**Figure 1:**
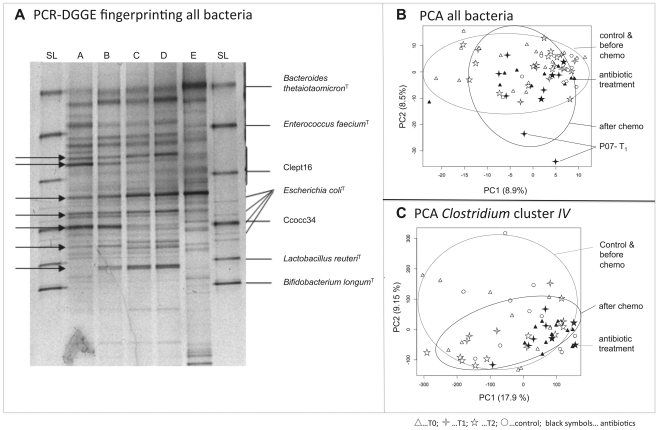
A PCR-DGGE fingerprinting of 16S rRNA coding regions of dominant bacteria over time. Bands that become stronger or nearly disappear following a single chemotherapeutic treatment are indicated with arrows. B Principal components analysis (PCA) based on dominant bacteria PCR-DGGE fingerprinting. The two outliers in the lower right corner of the plot are two samples of P07 following blood stem cell transplantation. C PCA illustrating the development of *Clostridium* cluster *IV* diversity in the course of chemotherapy and antibiotic treatment. Cluster *IV* diversity drops right after chemotherapy, causing a grouping of samples. Samples under antibiotic treatment (indicated as grey dots) group even closer, indicating a strong influence of antibiotics on *Clostridium* cluster *IV* diversity. A, sample of P01 before chemotherapy B, C and D, samples of P01 after chemotherapy; E, healthy control; SL, unrelated standard lane; black symbols… patients under chemotherapy and antibiotic treatment.

DGGE fingerprinting analyses of all bacteria, *Clostridium* cluster *IV* and *Clostridium* cluster *XIVa* indicate a highly diverse dataset between individuals and uniqueness of fecal microbiota. Table 1 shows the average number of bands in cancer patients at the three time points and for controls over all time points. It becomes apparent that the average number of bands within *Clostridium* cluster *IV* declined immediately after chemotherapy (T1), followed by a recovery at T2. The average number of *Clostridium* cluster *XIVa* bands decreased after onset of chemotherapy and remained low also at T2. The datasets were subjected to principal component analysis (PCA). PCA extracts underlying components of samples according to their variance. Figure 1A illustrates the bacterial fingerprints of sample P01 over time. Figure 1B displays the PCA analysis of all bacteria. Most samples taken after chemotherapy are grouped together with all other samples. Patients who receive antibiotics are indicated as black symbols. They cluster together with the samples taken after chemotherapy and also with the majority of samples before chemotherapy and healthy controls. There are two exceptions though: Two samples from P07 after chemotherapy under antibiotic treatment are outliers in the lower right part of the PCA plot. P07 received blood stem cell transplantation resulting in a sharp decline in bacterial abundances as measured with quantitative PCR. The first two principal components explain 17.4% of variance.

**Table 1:** Number of bands observed in PCR-DGGE fingerprinting in oncology patients before chemotherapy (T_0_), immediately after chemotherapy (T_1_) and 5–9 days after chemotherapy (T_2_) and healthy controls averaged over all time points.

Time point	All bacteria	*Clostridium* cluster *IV*	*Clostridium* cluster *XIVa*
T_0_	18.9±4.6	14±7.0	8±3.2
T_1_	19.7±4.9	10±6.0	4.9±3.6
T_2_	19.6±3.6	15±6.0	5.2±2.6
control	19.2±3.5	12.0±5.0	8.9±3.0

Figure 1C shows the principal components analysis of *Clostridium* cluster *IV*. DGGE fingerprints of individuals after chemotherapy are found to be less variable than healthy controls and patients before onset of treatment. Although overlapping, PCA resulted in grouping of band patterns before and after chemotherapy. Additional effects by antibiotic treatment became evident: Antibiotic treatment significantly reduced the diversity within the *Clostridium* cluster *IV* (p = 0.00003) with Shannon diversity index being 1.4±0.7 compared to patients under chemotherapy alone 2.1±0.6. In the PCA plot, samples affected by antibiotics are found in the lower right corner of the plot. This means that they are grouped according to their variance along principal component (PC) 1 and 2. These two PCs explain 17.9% and 9.15% of the variance in the dataset, underlining the validity of this interpretation. Principal components analysis of *Clostridium* cluster *XIVa* is not shown.

### Chemotherapeutic treatment with or without antibiotics decreases absolute bacterial numbers in comparison to healthy controls

**Figure 2:**
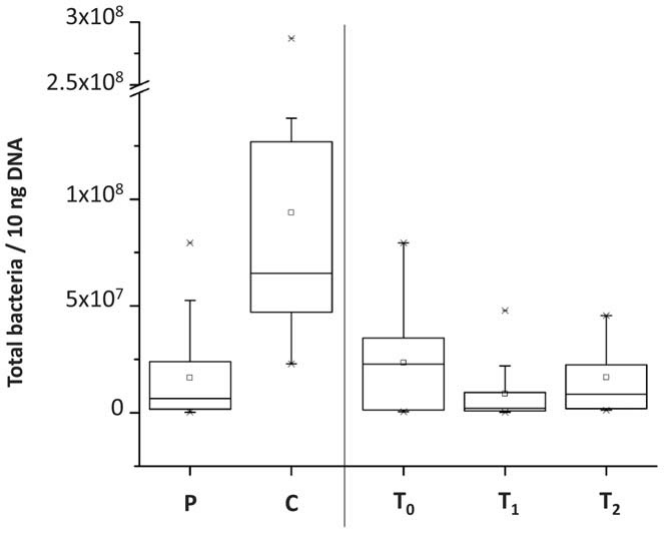
TaqMan qPCR quantification of bacterial 16S rRNA coding regions showing lower abundance in patients undergoing chemotherapy and antibiotic treatment (P) than healthy controls (C). T_0_, samples taken before a single shot of chemotherapy; T_1_, 1–2 days after chemotherapy; T_2_, 5–9 days after chemotherapy; Asterisk indicates a significant difference at p<0.05.

**Figure 3:**
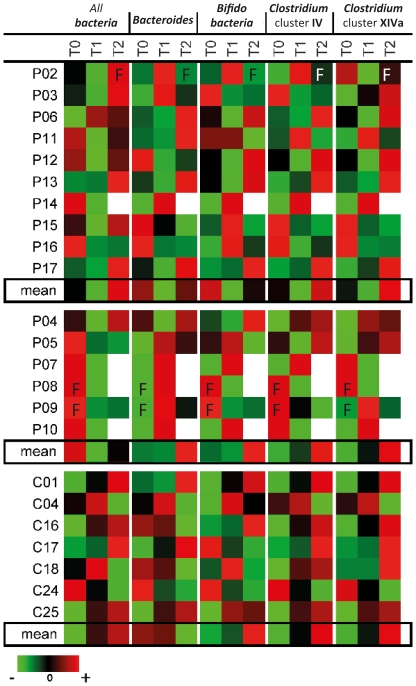
Abundances of bacterial 16S rRNA coding regions over time in oncology patients (P) and healthy controls (C). The declined abundances of bacteria, *Bacteroides*, *Clostridium* cluster *XIVa*, *Clostridium* cluster *IV* and bifidobacteria immediately after chemotherapy (T_1_) were observed to recover several days after treatment (T_2_). Patients P04, P08 and P13 had never received chemotherapy before; P04, P05, P07, P08, P09 and P10 took antibiotics. Values were z-scored for presentation in this heatmap showing changes over time rather than absolute abundances. T_0_, before chemotherapy; T_1_, 1–2 days after chemotherapy; T_2_, 5–9 days after chemotherapy; F, fever; S, blood stem cell transplantation.

To study whether chemotherapy with or without antibiotics changes the human GI microbiota composition in contrast to healthy individuals and over time, we investigated absolute numbers and relative percentages of bacterial subgroups. Absolute numbers give an indication about the direct antimicrobial effects of the treatments. Relative quantification is able to identify which bacterial subgroups are particularly affected and helps to describe the community disruption induced by chemotherapy with or without antibiotics. In absolute numbers, oncology patients harbored significantly less bacteria (p<0.05) than healthy control (figure 2). From already low bacterial counts before chemotherapy, bacterial abundance significantly declined further (p = 0.037) immediately after chemotherapy (T1) and recovered 5–9 days later (T2) in comparison to time points before treatment (T0). Absolute numbers of bacteria in different time points of healthy controls are following a lognormal distribution in contrast to microbiota abundances in oncology patients. The decrease in total bacteria following chemotherapy (p = 0.037) was significantly greater than any variation in copy numbers observed in healthy controls (p = 0.027). The observed decrease after chemotherapy affected the *Bacteroides* (p = 0.044), the bifidobacteria (p = 0.034) and *Clostridium* cluster *IV* (p = 0.049) as shown in figure 3. There were also fewer absolute numbers of *Clostridium* cluster *XIVa*, but this difference was not significant. All patients with fever showed an increase in total fecal microbiota (see figure 3). In sample P07 a sharp decline affecting all bacteria and bacterial subgroups was observed at T1 (figure 3), following blood stem cell transplantation and medical intervention.

Patients who received antibiotics had highest abundances of all bacteria (p = 0.000003) amongst all patients (data not shown). This bacterial overgrowth affected the *Bacteroides*, the bifidobacteria and *Clostridium* clusters *IV* and *XIVa*, since relative abundances of those subgroups did not stand out significantly. Thus, patients were grouped according to their chemotherapeutic cycle regardless whether or not they received antibiotics. The influence of antibiotics on the species composition as assessed with PCR-DGGE fingerprinting is discussed in the previous section.

### *Clostridium* cluster *XIVa* shows great alterations due to chemotherapeutical interventions, while the *Bacteroides* and bifidobacteria seem to be marginally affected

Relative quantification of *Clostridium* cluster *XIVa* as percentage of total bacterial DNA showed that oncology patients harbored significantly less *Clostridium* cluster *XIVa* (p = 0.047) than healthy controls. The mean proportion of *Bacteroides* in stool samples was 26±12% in chemotherapy patients and 22±14% in healthy individuals. The mean percentage of bifidobacteria in patients was 0.8±1.4% and 0.3±0.6 in controls. Patients harbored on average 16±9% of *Clostridium* cluster *IV* and 18±12% of *Clostridium* cluster *XIVa*, while controls harbored 20±12% and 24±15% of clostridial clusters *IV* and *XIVa*.

### *Clostridium* cluster *XIVa* higher before chemotherapy than after

The mean percentage of *Clostridium* cluster *XIVa* before chemotherapy was 22±13% compared to after chemotherapeutic cycles with 19±12%. The average amount of *Bacteroides*, bifidobacteria and *Clostridium* cluster *IV* were 26±11%, 1.4±2% and 16±9% at time points before chemotherapy and 28±14%, 0.5±1.2% and 18±12% after chemotherapy. Figure 3 illustrates the development of the microbiota in the course of antibiotic treatment. Data were normalized for clarity, so that changes in abundances from time point T0 (before onset of treatment) to T1 (1–4 days after chemotherapy) and T2 (5–9 days after chemotherapy) rather than relative abundances are shown. It can be seen that chemotherapy causes a dramatic reduction of microbiota abundance immediately after chemotherapy, affecting all subgroups. As mentioned above, the significant decrease in all bacteria following chemotherapy was significantly greater than any variation in copy numbers observed in healthy controls (p = 0.027).

### *C. difficile* colonization found in individuals receiving chemotherapeutic and antibiotic treatment

To find out whether the chemotherapeutic and antibiotic disruption favors the growth of pathogens, we investigated the abundance of *C. difficile*. Three out of seventeen patients receiving chemotherapy harbored *C. difficile* (data not shown). Patient P09 harbored C. difficile at all time points investigated. Mean proportion over all four samples of P09 was recorded as 0.4±0.7%, yet the highest level (1.22% of total bacteria) occurred at sampling point T1 immediately after chemotherapeutic and antibiotic treatment. *C. difficile* was detected in P11 (3.90% of all analyzed bacteria) after chemotherapeutic intervention at time point T1. P14 carried *C. difficile* in low abundance directly after onset of chemotherapy (0.003% of all analyzed bacteria). Samples of patients P09 and P11 at T0 and T1 were further analyzed in 454 sequencing.

### High throughput sequencing

**Figure 4:**
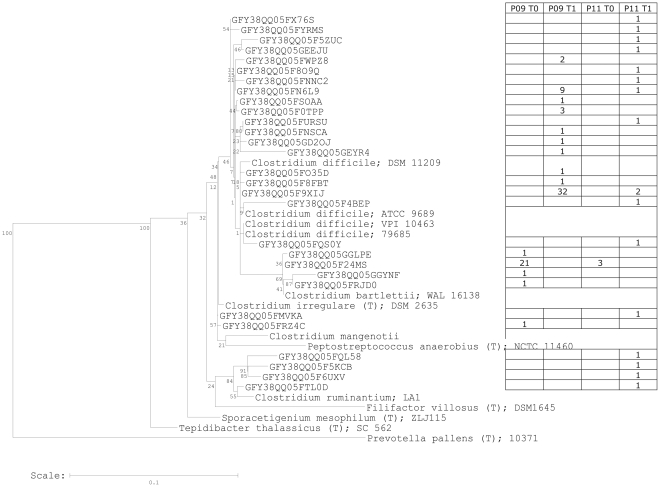
Phylogenetic tree showing the *Peptostreptococcaceae* found in samples from two oncology patients before and after chemotherapy. Identical sequences were grouped; the table on the right hand side shows their abundances in the 454 sequencing dataset. Sequences with >98.9% similarity to *Clostridium difficile* appeared only in samples taken immediately after chemotherapeutic cycles. Numbers indicate bootstrap values after 100 resamplings.

**Figure 5:**
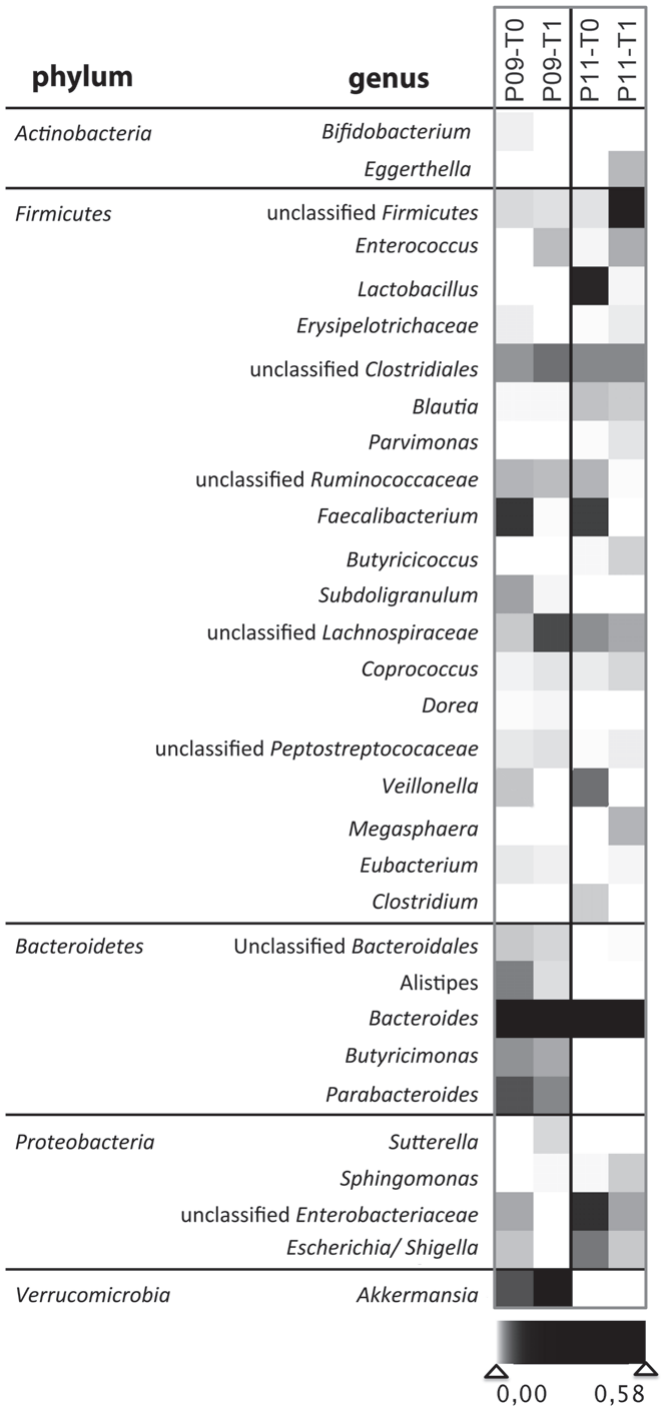
Heatmap showing abundances within the 454 sequencing dataset on the genus level. High throughput sequencing of samples P09 and P11 before (T_0_) and after therapy (T_1_) further helped to characterize the influence of a single chemotherapeutic cycle on the GI-microbiota. P11 was treated with chemotherapy alone and P09 also received antibiotic treatment.

High throughput sequencing showed a dramatic increase in sequences within the *Peptostreptococcaceae* towards sequences 98.9–100% similar to *C.difficile* ^T^ (figure 4): *Clostridium bartletti* related sequences (98.1–100% similarity) were only detected before chemotherapy (T0). After chemotherapy (T1), 63 sequences 98.9–100% similar to *C.difficile* appeared in samples P11 and P09. In accordance with the phylogenetic classification by the ribosomal database project, they are shown as ‘unclassified *Peptostreptococcaceae*’ in figure 5.

Furthermore pronounced reductions of *Faecalibacterium* spp. as well as lactobacilli, *Veillonella* spp., bifidobacteria (in P09) and *E.coli*/*Shigella* became apparent in response to chemotherapy (figure 5). The abundance of lactobacilli decreased in both patients after chemotherapy, in P09 from already low levels. Individual P11 did not receive concomitant antibiotics, whereas P09 did. In P09 and P11 *Faecalibacterium* spp. decreased dramatically from 9.5% and 8.3% to 0.07% and 0.00%, respectively. In both individuals *Enterococcus faecium* increased following chemotherapy. Furthermore, less abundant sequences appeared that were attributable to bacterial genera not detected before chemotherapy. These genera are: *Eggerthella*, *Megasphaera*, *Parvimonas* (only in P11), *Anaerostipes*, *Eubacterium*, *Anaerococcus*, *Methylobacterium*, *Holdemania*, *Turicibacter*, *Akkermansia*, *Sutterella* (only in P09), *Sphingomonas*, *Anaerotruncus*, *Coprococcus*, *Streptococcus* and *Dorea*. Species with abundance <0.01% of all sequences are not shown in figure 5. The number of *Blautia* species from *Clostridium* cluster *XIVa* remained constant in the 454 sequencing datasets before and after chemotherapy.

## Discussion

Chemotherapeutic and antibiotic use is associated with severe side effects such as mucositis, diarrhea and constipation. These side effects increase the cost of health services and are often life threatening [22]. Chemotherapeutic and antibiotic treatment has a detrimental impact on the host microbial ecosystem, which is important for host mucosal protection [19] and thereby increases the risk of infection [17]. Overgrowth of species with potential pathogenicity such as toxigenic *C. difficile* and inflammatory complications are among the most common serious complications of chemotherapy and antibiotic treatment among patients with cancer [15], [17].

We investigated how the use of cancer chemotherapy (in some individuals together with antibiotic treatment) perturbs the fecal microbial ecosystem during the course of therapy. We assessed if the microbiota is able to return to its original profile after chemotherapeutic and antibiotic intervention with special interest in the abundance of *C. difficile*. We used a combination of molecular methods including high-throughput sequencing to compare diversity (PCR-DGGE) and abundance (qPCR) of all bacteria, *Bacteroides*, bifidobacteria, *Clostridium* cluster *IV*, *Clostridium* cluster *XIVa* and *C. difficile* between groups and different time points of chemotherapy. The majority of previous studies on the effect of chemotherapy on human fecal microbiota used standard microbiological culture techniques [16], [22]. Other studies have focused on the colonization of pathogenic bacteria [17], [25] in patients with cancer and chemotherapy-induced diarrhea [22], [26]. As mentioned above, we used feces as source of information. Fecal microbial communities are composed of autochthonous gut members and transient bacteria. Even though the fecal microbiota might be different from the adherent microbiota, we chose fecal samples to investigate the microbial composition of the intestinal microbiota because they are easy to collect, are less invasive and reflect shifts in microbial population composition [11].

In this study, we assessed species richness using PCR-DGGE fingerprinting. Each lane of a PCR-DGGE gel represented a microbial fingerprint of a fecal sample; each band within a lane corresponded to one bacterial species, although different species may sometimes be represented by the same band [17]. It has also been observed that one bacterial strain may form several bands due to multiple 16S rRNA operons, e.g. *E.coli* (figure 4A). The limitations of DGGE in microbial analysis have been previously described [27]. Nevertheless, substantial information about species composition can be obtained from very complex microbial communities such as the gut microbiota [27]. We found decreased species richness immediately after the chemotherapeutic shot, especially within *Clostridium* cluster *IV* where the number of different bands decreased from 14±7 before chemotherapy (T_0_) to 10±6 bands shortly after (T_1_). The microbiota recovered to a richness of 15±6 *Clostridium* cluster *IV* bands per individual, but at a different composition, as evidenced by the grouping of samples in principal components analysis.

For quantification of fecal microbiota we used the strains *Bacteroides thetaiotaomicron^T^*, *Bifidobacterium longum* ssp. *longum^T^* and *C. difficile* as well as the clones CL16 and CC34 as standards. However, a mixture of different strains for qPCR standards might result in a more accurate image of the human microbiota. Therefore absolute amounts should be considered as semi-quantitative.

Grouping oncology patients with and without antibiotic treatment poses a risk to falsely interpret the effects of antibiotic treatment as effects of chemotherapy. Patients who received antibiotics had significantly higher bacterial abundances than patients without antibiotics. This observation might be the reason for antibiotic treatment rather than its effect [24]. The abundance of bacterial subgroups, also *Clostridium* cluster *IV*, changed together with total bacteria both in patients with and without antibiotics. The sharp reduction of bacteria immediately after chemotherapy equally affected patients with and without antibiotics. In PCR-DGGE analysis we found that the all bacteria and *Clostridium* cluster *XIVa* fingerprints did not differ significantly in patients *with* or *without* antibiotics. This indicates that the use of antibiotics does not fully explain the observed changes. Previous work [17] has also found additional effects of chemotherapy in cases under prophylactic antibiotic treatment. Although the *Clostridium* cluster *IV* abundance did not differ significantly due to antibiotics, PCR-DGGE fingerprints showed grouping of patients under antibiotic treatment in principal components analysis.

Despite high individual variations, we show a significantly lower absolute bacterial load in feces of patients receiving chemotherapy in comparison to healthy controls. These findings are in line with data from van Vliet *et al.* (2009) who reported 100-fold lower total bacterial numbers during chemotherapy than in healthy controls.

The abundance of fecal microbiota decreased after a single cycle of chemotherapy. After the end of chemotherapeutic administration the bacterial abundance recovered within a few days, sometimes even showing a “rebound-effect” with numbers elevating above initial levels. Relative numbers of *Clostridium* cluster *IV* and *XIVa* showed great alterations due to chemotherapeutical interventions, while the bifidobacteria seemed to be less affected. In agreement with previous results [16] increased counts of *Bacteroides* spp. were found in patients undergoing chemotherapy. Nyhlèn *et al.* (2007) also reported significant increases in yeast in patients, making it a focus for further research in immunocompromised patients. Samples taken immediately after chemotherapy had a lower diversity within *Clostridium* cluster *IV*. Antibiotics strongly contributed to the reduced diversity of cluster *IV* but were not alone responsible for this effect. A few days later we observed a quantitative recovery, but not a recovery of the composition as evidenced by clustering of DGGE fingerprints.

The incidence of *C. difficile* in subjects P09 and P11 immediately after chemotherapy is accompanied by a decrease of the genera *Bifidobacterium*, *Lactobacillus* and *Clostridium* cluster *IV*. Sequences attributable to *Faecalibacterium prausnitzii* decreased dramatically from 9% to zero. The anti-inflammatory *F. prausnitzii* was associated with dietary fiber in colonic fermentation of healthy subjects [28] and found at low abundance in individuals suffering from inflammatory bowel diseases [29] [30], [31]. *Enterococcus faecium* increased following chemotherapy, possibly filling the ecological niches vacated by the lactobacilli and bifidobacteria. *Enterococcus faecium* is a facultative pathogenic bacterium causing life-threatening infections especially in nosocomial settings [32]. *Enterococcus faecium* has previously been found to increase in wastewater upon treatment [33]. The acquisition of multi-resistant *E. faecium* strains has been described in hospital environments under high selective antibiotic pressure. Under such conditions probiotic strains were demonstrated as unable to prevent nosocomial infection [34].

After chemotherapy less abundant sequences appeared that were not detected before treatment. These genera are: *Eggerthella*, *Megasphaera*, *Parvimonas*, *Anaerostipes*, *Eubacterium*, *Anaerococcus*, *Methylobacterium*, *Holdemania*, *Turicibacter*, *Akkermansia*, *Sutterella*, *Sphingomonas*, *Anaerotruncus*, *Coprococcus*, *Streptococcus* and *Dorea*. *Eggerthella lenta* was described to convert dietary lignans to the bioactive enterolactone [35]. *Megasphaera* spp. have been described as propionate-producers that utilize lactate [36] comparable to *Veillonella* spp. that were no longer detected by 454 sequencing after chemotherapy. The butyrate-producing *Anaerostipes caccae* and *Eubacterium hallii* utilize lactate as well. They were suggested to compete for lactate with sulfate-reducing bacteria such as *Desulfobacter piger* whose preferred co-substrate is lactate. High concentrations of sulfate are toxic for the gut epithelium and may contribute to bowel disease [37]. Microorganisms of the genus *Methylobacterium* are facultative methylotrophic, gram-negative rods that are ubiquitous in nature and rarely cause human disease, except in subjects with pre-existing immunosuppression. For instance, in 2010, a case of *M. fujisawaense* infection was described in a patient with relapsed acute leukemia undergoing unrelated allogeneic hematopoietic stem cell transplantation [38]. *Turicibacter* is a poorly known genus previously found in weaned piglets, known to be susceptible to chlortetracycline [39]. In humans, *Turicibacter* spp. have been found in the ileal pelvic pouch of a former ulcerative colitis patient [40]. *Akkermansia muciniphila* is a common mucin-degrading bacterium of the human GI. Its prevalence has been described to be 10^8^ cells/g feces in adults, decreasing with age [41]. *Dorea* spp. are mucosa-associated bacteria of the human GI that are members of the *Clostridium coccoides* rRNA group of organisms [42], [43].

Further research is needed to elucidate if there is a causal relationship between growth of *C. difficile* and decreased abundance of lactobacilli, bifidobacteria and *Clostridium* cluster *IV*, especially the anti-inflammatory *Faecalibacterium prausnitzii*. The increase of mucus-degrading bacteria might be a result of *C.difficile* and probably also *E. faecium* associated inflammation of the gut epithelium. Mucus hypersecretion is a common symptom of irritable bowel syndrome, ulcerative colitis and bacterial infections of the gut epithelium [44], [45]. The lactate-utilizing microbiota shifted from *Veillonella* spp. to *Anaerostipes*, *Eubacterium* and *Megasphaera* spp. This change may be interpreted as a beneficial adaptation, because lactate could otherwise be used as a co-substrate for sulfate-reduction. Sulfate-reducing bacteria, however, were not detected here.

The oncology patients assessed here suffered from a variety of malignancies and received different chemotherapy treatment regimes. Only two participants (P01 and P08) had never received any cancer therapy before, while all others had a history of chemotherapeutic treatment. Therefore, the observed changes are likely to be influenced by previous cycles of chemotherapy. For example, the significantly lower bacterial abundance in cancer patients before chemotherapy in comparison to control could be a consequence of previous treatments. The results presented here illustrate changes due to a single chemotherapeutic cycle, but cannot rule out, that these changes occurred as a consequence of several chemotherapeutic cycles over the course of several years. Six cancer patients also received antibiotics. These patients were characterized by significantly elevated abundances of bacteria. This finding confirms the diagnosis ‘bacterial infection’ for which antibiotic treatment was prescribed. *Clostridium* cluster *IV* PCR-DGGE profiles revealed a shift in species composition by chemotherapy, and even more so by antibiotics. Thus we conclude that antimicrobial treatment significantly reduces the species richness of the *Clostridium* cluster IV, with the anti-inflammatory *Faecalibacterium prausnitzii* being the most abundant representative. Van Vliet *et al.* (2009) tested the effect of chemotherapy *in vitro* and showed a direct bacteriostatic effect of chemotherapeutics on bacterial growth.

Further research is needed to show whether changes in bacterial colonization play a role in the development and maintenance of mucosal barrier function, infection and inflammation [17].

The use of prebiotics, probiotics and bacterial products, such as butyrate to prevent mucosal barrier injury and its complications could be a promising concept in restoring impaired functions or enhancing specific desirable functions of the microbiota. The use of pre- and probiotics to affect the composition and metabolic activity of the fecal microbiota in times of cancer chemotherapy and immunosuppression might be part of future research.

In conclusion, chemotherapy treatment causes changes in fecal microbiota, which coincide with the development of *C. difficile* infection in some patients. These changes in microbiota may have systemic effects and may contribute to the development of chemotherapy-induced mucositis, influencing important beneficial functions of the microbial ecosystem.

## Materials and Methods

### Ethics statement

The Viennese Human Ethics committee (3., Thomas-Klestil-Platz 8/2) under the chair of Dr. Karin Spacek, approved the proposal of the project “Analysis of microbiota in feces of patients with immunosuppression”. Votum: EK 07-153-VK, 2008. From all participants involved in the study written consent was obtained.

### Study participants and study design

Seventeen subjects receiving ambulant chemotherapy with or without antimicrobial therapy (aged 59±13 y, BMI 27±6) from the Sozialmedizinisches Zentrum Ost (SMZ Ost) in Vienna and seventeen healthy individuals (aged 65±18 y, BMI 24±5) joined this study. Four fecal samples within two weeks were collected of each ambulant oncology patient in order to collect samples before and after a single immune-suppressive chemotherapy cycle. The four samples obtained from every patient were grouped into three groups: samples taken before the day of chemotherapy (T0), samples taken 1–4 days after chemotherapy (T1) and samples taken 5–9 days after chemotherapy (T2). Healthy individuals also donated four samples during two weeks. Gender ratio among healthy controls was 56% female, 44% male. Oncology patients were 47% female and 53% male. Three out of seventeen patients (P04, P08, P13) had never received any chemotherapy before, while the others had a history of chemotherapy. Anonymous medical records reported types of malignancies as well as chemotherapeutic and antimicrobial treatment as shown in table 2.

**Table 2:** Relevant clinical data of study participants undergoing immunesuppressive chemotherapy.

name	diagnosis	chemotherapeutic treatment	antimicrobial treatment	other condition
P01	urothel carcinoma	gemcitabine, cisplatinum		
P02	plasmocytoma, multiple myeloma	bortezomib, dexamethasone		rheumatismfever at 4^th^ sampling
P03	Non-Hodgkin lymphoma	bendamustine		diabetes II, adipositas, hypertension
P04	ovarian fibroma	taxol, carboplatin	levofloxacin	
P05	multiple myeloma	bortezomib, doxorubicin, dexamethasone	cotrimoxazole	osteoporosis
P06	mamma carcinoma	pegylated liposomal doxorubicin hydrochloride, gemcitabine		
P07	Non-Hodgkin lymphoma	high dose radiation therapy and PBSCT	cotrimoxazole, piperacillin, tazobactam	fever at 2^nd^ and 3^rd^ sampling
P08	monozytic leukemia	cytarabine, idarubicin	cotrimoxazole, piperacillin, tazobactam	fever at 2^nd^ sampling
P09	acute leukemia	high dose Ara-C, radiated erythrocyte concentrate	piperacillin, tazobactam	fever at 2^nd^ sampling
P10	Non-Hodgkin lymphoma	ifosamid, etoposid, methotrexat	levofloxacin	
P11	Acute lymophoblastic leukemia	cytarabine, methotrexat		adipositas, hypertension
P12	small intestinal tumor	cetuximab		
P13	rectal tumor	capecitabine, oxaliplatin		
P14	thymus tumor	taxol, carboplatin, bevacizumab, radiation		
P15	Acute lymophoblastic leukemia	cyclophosphamide, methotrexate, doxorubicin, cytarabine, vincristine		diabetes II
P16	Acute lymophoblastic leukemia	cytarabine, mitoxantrone		
P17	colon tumor	oxaliplatin, capecitabine, bevacizumab, irinotecan, monoclonal antibodies		

PBSCT… peripheral blood stem cell transplant.

We interviewed all study participants assessing age, gender, body length, weight, health status (chronic and acute diseases), and life-style aspects such as alcohol consumption and physical activity. Dietary habits were assessed using a food frequency questionnaire. Exclusion criteria for healthy controls were (a) antimicrobial medication (b) chemotherapeutic treatment and (c) pre- and probiotics at least three months before sample collection. Approval for this study was obtained from the Viennese Human Ethics committee (3., Thomas-Klestil-Platz 8/2).

### Stool sample processing

After collection, study participants immediately stored their samples at -18°C in their homes. Stool samples were still frozen when brought to the laboratory and then immediately stored at −70°C. A 200 mg aliquot of each sample was treated twice for 45 s in a bead-beater (Mini-Beadbeater-8). Thereafter DNA was extracted using the QIAamp® DNA Stool Mini Kit (QIAGEN) following the manufacturer's protocol. The DNA was stored at −20°C until analysis.

### Type strains

We used type strains, known to be part of the human gastrointestinal microbiota and cloned sequences to design a DGGE standard lane marker. Type strains *Bacteroides thetaiotaomicron* DSM 2079T, *Enterococcus faecium* DSM 20477T, *Lactobacillus reuteri* ATCC 55730T, *Bifidobacterium longum* ssp. *longum* DSM 20097T, *Escherichia coli* IMBH 252/07 and clones CL16 and CC34 (see below) were used for creating a comparable standard lane marker for DGGE gels analyzing all bacteria. *E.coli* IMBH 242/07 gave 4 bands due to its multiple operons for the 16S rRNA gene.

### Clone library

To create a standard lane marker for DGGE analysis and to identify members of the *Clostridium* cluster *XIVa* we constructed clone libraries from two stool samples of healthy volunteers. For this purpose PCR products amplified with primers 195-F [46] and Ccocc-R [47] were inserted into a p-GEM Easy Vector (Promega) following the instructions of the manufacturer. Nucleotide sequences were corrected for primer and vector sequences in CodonCodeAligner (www.codoncode.com) and taxonomically identified using the online tools of the ribosomal database project (http://rdp.cme.msu.edu/). The clone library used for creating a standard lane marker for DGGE analysis of *Clostridium* cluster *IV* has previously been described [48]. Clones CL16 (*Clostridium leptum* 16) and CC34 (*Clostridium coccoides* 34) were also used as positive controls in Taqman qPCR.

### Polymerase chain reaction (PCR)

PCR was carried out amplifying 16S rRNA gene sequences from bacteria in fecal samples, type strains and cloned sequences for DGGE analysis as well as for creation of the clone library using group- and kingdom- specific primers (table 3). The PCR reaction mixture consisted of ready-to-use mastermix (Promega) with 1.5 mM MgCl2, 500 nM of primers and 2 µl of template DNA. When amplifying fecal samples, bovine serum albumin (Fermentas) was added to a final concentration of 400 µg/ml. We used a Robocycler (Stratagene) for all amplifications.

**Table 3:** 16S rRNA gene primers used for PCR-DGGE fingerprinting.

Target organism	Primer	Sequence (5′-3′)	Ann. temp (°C)	Reference
All bacteria	27f	GTGCTGCAGAGAGTTTGATCCTGGCTCAG	57	[52 T., Blocker 1989]
	985r	GTAAGGTTCTTCGCGTT	57	[53]
	341f-GC	CCT ACG GGA GGC AGC AG	55	[49]
	518r	ATT ACC GCG GCT GCT GG	55	[54]
*Clostridium* cluster IV	sg-Clept-F-GC	GCA CAA GCA GTG GAG T	55	
	sg-Clept-R3	CTT CCT CCG TTT TGT CAA		[55]
*Clostridium* cluster *XIVa*	Ccocc-F-GC	AAATGACGGTACCTGACTAA	55	
	Ccocc-R	CTTTGAGTTTCATTCTTGCGAA		[55]
GC-clamp		CGCCCGGGGCGCGCCCCGGGCGGCCCGGGGGCACCGGGGG		[49]

### PCR-DGGE-fingerprinting

DGGE was performed as previously described [49]. Primer pairs and annealing temperatures to analyze the diversity of (a) all bacteria, (b) *Clostridium* cluster *IV* and (c) *Clostridium* cluster *XIVa* are described in table 3. PCR products were separated by polyacrylamide gels with a denaturing gradient of 30–60% for predominant bacteria, 30–50% for *Clostridium* cluster *IV* and 35–50% for *Clostridium* cluster *XIVa*. Electrophoresis was performed for 9 h at 129 V at 60°C (predominant bacteria), 5 h at 200 V at 60°C (*Clostridium* cluster *IV*) and 7 h at 200 V at 60°C (*Clostridium* cluster *XIVa*). Standard lane markers were created for each DGGE analysis assay to ensure reliable gel-to-gel comparison. These standard lane markers (described above) were loaded in triplicate on each gel to adjust for gradient-variations between gels. We analyzed PCR-DGGE fingerprints using GelComparII (www.applied-maths.com). When generating the band comparison, a 1% tolerance was selected. Principal components analysis (PCA) was applied on quantitative band comparison datasets in ‘R’ (www.r-project.org) using the default settings. Shannon diversity index was calculated on quantitative band information as well with the default settings implemented in the ‘vegan’ package in ‘R’ (www.r-project.org). Shannon index is defined as H = −∑ pi ln pi, where pi is the proportional abundance of species i. In short, the higher the Shannon index is, the higher is the diversity. For interpretation of results, samples were grouped into three groups: samples taken before the day of chemotherapy (T0), samples taken 1–4 days after chemotherapy (T1) and samples taken 5–9 days after chemotherapy (T2).

### Quantitative TaqMan qPCR

The abundance of bacteria and bacterial subgroups was measured by 16S rRNA gene-targeting TaqMan qPCR in a Rotorgene 3000 (Corbett Life Science). Primers, annealing temperatures and expected product sizes are shown in table 4. Each sample was analyzed in duplicate. Amplifications were carried out in a total volume of 10 µl consisting of 5 µl Taq-Man SensiMix DNA Kit (Quantance), 1 µl of each primer and Taq-Man probe (concentrations see table 4) and 10 ng of bacterial DNA. Amplification programs included an initial denaturation at 95°C for 10 min followed by 40 cycles consisting of denaturation at 95°C for 30 s, annealing at 55°C (all bacteria, *Clostridium* cluster *IV*), 56°C (*Clostridium* cluster *XIVa*), 58°C (*C. difficile*) or 60°C (*Bacteroides*, bifidobacteria) for 30 s and extension at 72°C for 50 s.

**Table 4:** Primers and probes used for TaqMan qPCR quantification of 16S rRNA genes.

Target organism	Primer and probe	Sequence (5′ - 3′)	Size (bp)	Conc. (nM)	Reference
*Bifidobacterium* spp.	Forward primer	GCG TGC TTA ACA CAT GCA AGT C	125	300	
	Reverse primer	CAC CCG TTT CCA GGA GCT ATT		300	
	Probe	(FAM)- TCA CGC ATT ACT CAC CCG TTC GCC -(BHQ-1)		150	[56]
*Bacteroides*	AllBac296f	GAG AGG AAG GTC CCC CAC	106	300	
	AllBac412r	CGC TAC TTG GCT GGT TCA G		300	
	AllBac375Bhqr	(FAM)-CCA TTG ACC AAT ATT CCT CAC TGC TGC CT-(BHQ-1)		100	[53]
All bacteria	BAC-338-F	ACT CCT ACG GGA GGC AG	468	1000	
	BAC-805-R	GAC TAC CAG GGT ATC TAA TCC		1000	
	BAC-516-P	(FAM)-TGC CAG CAG CCG CGG TAA TAC-(BHQ-1)		200	[50]
*Clostridium* cluster *IV*	sg-Clept-F	GCA CAA GCA GTG GAG T	239	400	
	sg-Clept-R3	CTT CCT CCG TTT TGT CAA		400	[47]
	Clept-P^++^	(FAM)-AGG GTT GCG CTC GTT-(BHQ-1)		200	This study
*Clostridium* cluster *XIVa*	195F	GCA GTG GGG AAT ATT GCA		500	[46]
	CcoccR	CTT TGA GTT TCA TTC TTG CGA A		500	
	CcoccP	(6-FAM)-AAATGACGGTACCTGACTAA-(BHQ-1)		150	[47]
*Clostridium diffficile*	CdiffF	TTG AGC GAT TTA CTT CGG TAA AGA		1000	[56]
	CdiffR	TGT ACT GGC TCA CCT TTG ATA TTC A	151	1000	
	CdiffP	(6-FAM)-CCA CGC GTT ACT CAC CCG TCC G-(BHQ-1)		200	

We used tenfold serial DNA dilutions of type strains *Bacteroides thetaiotaomicron*T, *Bifidobacterium longum* ssp. *longum* ^T^ and *C. difficile* as well as cloned sequences and one fecal sample to construct standard curves for comparison of PCR reaction efficiencies among different experiments.

We quantified DNA of *Bacteroides thetaiotaomicron* ^T^, *Bifidobacterium longum* ssp. *longum* ^T^ and C. difficile, using the nanodrop method and calculated DNA copies/µl through mean G+C content of each strain. Clones CL16 and CC34 were amplified with the SP6 Promoter Primer (Promega, Cat.# Q5011) and the T7 Promoter Primer (Promega, Cat.# Q5021) and the PCR product quantified using a nanodrop machine. Knowing the sequences of these two PCR products and their flanking vector sequences we could quantify the copy numbers and use it as standards. Relative percentages of bacterial subgroups were calculated in relation to total rRNA gene copies amplified with primer pair BAC-338-F and BAC-805-R [50].

We reviewed sensitivity of PCR reactions with stepwise dilutions of standard curve DNA until we achieved sensitive detection levels of PCR. The specificity was confirmed using non-target DNA.

### High throughput sequencing

In total, four samples (P09-T0, P09-T1, P11-T0, P11-T1) were amplified with primer 525F (5′- TCAGCAGCCGCGGTAATAC -3′) and 926R (5′-TCCGTCAATTCCTTTGAGTTT -3′) using a high-fidelity DNA polymerase (Phusion®, Finnzymes, Thermo Fisher Scientific) and submitted to 454 barcode sequencing (AGOWA, Berlin, Germany), resulting in a total of 113 000 reads. The sequences were trimmed and aligned using the pyro pipeline of the ribosomal database project (http://rdp.cme.msu.edu/). Only sequences longer than 150 bp were retained, resulting in 3886 to 6811 sequences per sample with the average lengths of 366 to 368 bp. All analyses were performed using the online tools of the ribosomal database project pyro pipeline (http://rdp.cme.msu.edu/). Results of the phylogenetic classification are shown as a heatmap [51]. The *Peptostreptococcaceae*, harbouring also *C.difficile*, from all four datasets were analyzed in more detail using the online tools of the ribosomal database project. 100% similar sequences were grouped and their abundances shown together with a phylogenetic tree.

### Data analysis

Statistical evaluation of differences between groups (chemotherapy and control) and changes within the chemotherapy group (all time points before and after chemotherapy) was carried out using the OriginPro version 8 (OriginLab, Northampton, MA). For two group comparisons of independent ordinal and interval values we used the two-sample t-test and the nonparametric Mann-Whitney U-test. For the analysis of related data we used the paired sample t-test or the non-parametric Wilcoxon signed-rank test. P values <0.05 were considered statistically significant. To show the decline in abundance immediately after chemotherapy qPCR results were plotted in heatmaps [51]. Values were z-scored for presentation in this heatmap showing changes over time rather than absolute abundances.

### Dietary aspects

We assessed the participants' dietary habits using a food frequency questionnaire. All study participants (patients and controls) were omnivores and showed similar consumption patterns of liquids, alcohol, fruits, vegetables, grains and milk products. Healthy controls stated more frequent consumption of fruits, whole grain products and alcohol several times a week compared to patients receiving chemotherapy.
